# Gross and Morphometric Anatomy of the Male Reproductive System of Bats (*Eidolon helvum*)

**DOI:** 10.1155/2014/358158

**Published:** 2014-03-26

**Authors:** A. Danmaigoro, J. E. Onu, M. L. Sonfada, M. A. Umaru, S. A. Hena, A. Mahmuda

**Affiliations:** ^1^Department of Veterinary Anatomy, Faculty of Veterinary Medicine, Usmanu Danfodiyo University, P.M.B 2346, Sokoto, Nigeria; ^2^Department of Theriogenology and Animal Production, Faculty of Veterinary Medicine, Usmanu Danfodiyo University, P.M.B 2346, Sokoto, Nigeria; ^3^Department of Veterinary Parasitology and Entomology, Faculty of Veterinary Medicine, Usmanu Danfodiyo University, P.M.B 2346, Sokoto, Nigeria

## Abstract

The present study aimed at examining the gross and morphometry of the reproductive tract of the male bats (*Eidolon helvum*). Thirty male bats (adults *n* = 17 and juveniles *n* = 13) were captured using net, weighed, aged using relative ossification of the wing bone, and dissected for gross examination. Morphologically, the mean body weight and forearm length in both adults and juveniles were 235.31 ± 6.30 g, 12.14 ± 0.19 cm and 69.54 ± 7.68 g, 7.77 ± 0.29 cm, respectively. The testicles were completely descended in adults with the penis projected cranially. The epididymides were found at the median border of the testis and continues as vas deferens. No significant differences (*P* > 0.05) were observed between right and left testicular weights in both adults and juveniles and also in lengths of different parts of the reproductive segments in both age groups assessed, respectively. This work has documented the gross anatomy of the male reproductive tract in bats. Ultrastructure and histochemistry are recommended for further insight into the reproductive biology.

## 1. Introduction

Bats (Chiroptera) are among the most diverse and widely distributed groups of mammals and can be found in all continents except in Antarctica [1]. There are around 1,150 species of bats. Bats are second to rodents in terms of numbers of genera and species, which represent nearly a quarter of all the species of mammals on earth with majority living in tropical and semitropical regions [1].

Bats are the only flying mammals and they have a wide range of feeding and roosting habits, social behaviours, and reproductive strategies [2]. Their nocturnal habits and diversity in their biology not only make bats a fascinating group of animals to study but also a difficult one.

The diversity and abundance of bats are probably attributable to a number of features, such as their biology, that are unique and most of their life tracks do not conform to typical mammalian patterns [3].* Eidolon helvum* is commonly found in moist and dry tropical rain forest [4].

The gonads and excretory structures embryologically develop from the intermediate mesoderm of the hindgut, with the gonadal ridge being formed as the precursor to gonad development [5]. The gonads arose as ridge-like thickening which in turn became thicker as the gonad differentiates to the primordial germ cells of the gonads [6].

The reproductive tract of male mammals usually consists of the testes and associated epididymis, vasa deferentia, accessory sex gland complex, urethra, and penis. The accessory sex gland complex consists of ampullary, prostate, urethra and Cowper's glands, and seminal vesicles [7]. However not all glands are present in every mammalian order [8] and in the Chiroptera as revealed by Bernard [9] on* Rhinolophus capensis.*

In recent years, bats have been implicated in numerous emerging infectious diseases and are increasingly recognized as important reservoir hosts for viruses that can cross species barriers to infect humans and other domestic and wild mammals [10]. Bats also have high ecological and economic importance due to their role in seed dispersal and serve as source of protein when taken as bush meat.

The gross anatomy of this system varies widely among different mammalian species [11]. Thus, variations exist between species in terms of the position of their reproductive structures. Some works on the physiology, breeding, and pathology of reproductive organs of the female bats [12–16] have been reported in different countries including Nigeria. Good understanding of the reproductive biology particularly that of the male reproductive system is very vital. Although* E. helvum *is found widely distributed in Northwestern Nigeria, little is known about its male reproductive morphology. This work is aimed at studying the gross and morphometry of the male reproductive tract of the bat (*Eidolon helvum*).

## 2. Materials and Methods

### 2.1. Source of Bats

A total number of thirty (30) male bats were used for the study (adult *n* = 17 and juvenile *n* = 13). Bats were sourced from Zuru town, Kebbi State in Northwestern Nigeria. The capturing process involved the use of hand nets with extendable poles, during daytime at the roosts of the bats. The bats were held in plastic cages with leaves hung on the ceilings of the cages to provide roosts for the bats and transported to the laboratory.

### 2.2. Bat Identification

Species were identified at the Zoological Laboratory Unit, Department of Biological Sciences, Usmanu Danfodiyo University, Sokoto, Nigeria. The following features were used for identification of the species as described by [17]. Males are usually larger than females and are bright orange in colour while females are usually yellowish in colour. The wings are long and tapered at the tip. The cheeks are also large and pouch-like. The neck and back displayed yellow colour, with the ventral side of the body being brown or grey. The head is large and pointed with white facial markings.

### 2.3. Age Estimation

Age estimation was achieved using relative ossification of the wing bones (4th metacarpal joint and phalanges) by transilluminating the wing of the bats using torchlight to visualize the cartilaginous zone of the long phalanges while the wing of the bat is being spread over a transparent solid plastic sheet lightened from below, so as to distinguish between juveniles and adults as described by [18].

### 2.4. Dissection

Following anaesthesia using chloroform (Prolabo, May, and Baker, Nigeria), the bats were sacrificed by severing the jugular vein and then placed on dorsal recumbency and a midline incision was made, extending from the xiphoid cartilage to the pubic symphysis. The peritoneum was reflected and intestines were displaced to gain access to the reproductive tract as described by [19]. Organs were examined in situ and exteriorized. Gross features of the reproductive tract were carefully examined and recorded. The length, width, circumference, and weight of the reproductive organs were measured using ruler, thread, and weighting balance (Shimadzu AW320, Germany), respectively. Photographs of the reproductive structures were taken using a digital camera (Canon, S1 S3, Tokyo, Japan, 7.5 megapixels).

### 2.5. Data Analysis

The data obtained were subjected to statistical analysis using InStat statistical package, version 3.05 (2000), in which descriptive statistic and Student *t*-test were used to check the significant differences between the weights of the testes and length of the structures (testes, epididymides, vas deferens, and penis) with ages and paired structures, where the probability of <0.05 was considered to be significant.

## 3. Results

The average body weights and mean forearm length of 235.31 ± 6.30 g and 12.14 ± 0.19 cm were obtained in the adult while 69.54 ± 7.68 g and 7.7 ± 0.29 cm were obtained in the juvenile bats as shown in Table 1.

**Figure 1:**
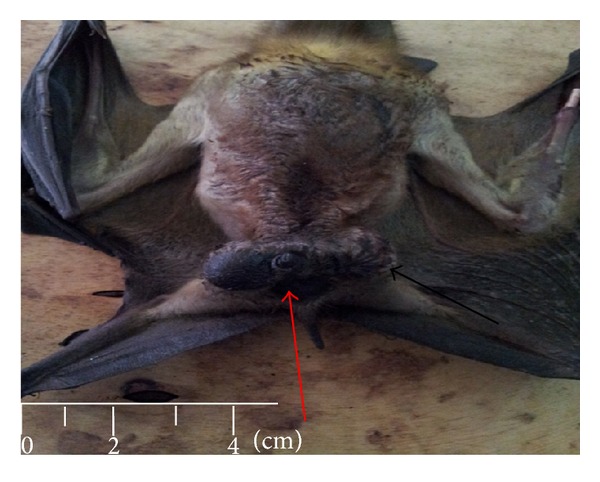
Adult male bat showing the external genital. Penis (red arrow) and the scrotum (black arrow).

**Figure 2:**
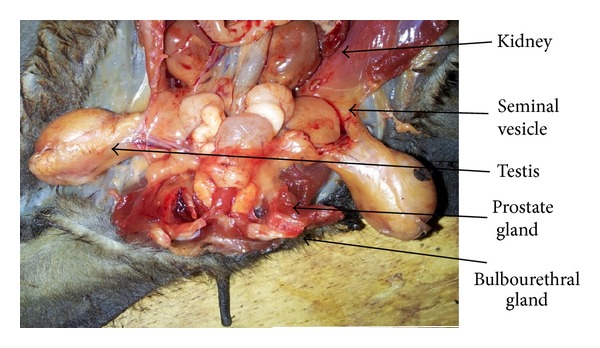
Male bat reproductive tract with accessory sex glands.

Just like other species, the bats were found to have paired testes, epididymis, and vas deferens with penis as shown in Figures 1 and 2. The testes are paired and migratory and seen outside the abdominal cavity, between the skin and muscles near the crest of the pubis lateral to the base of the penis. The testes were completely descended into the scrotum in adult bats and were ovoid shaped and milky-white in colour (Figures 1 and 2). The testes were covered with skin and enclosed by strong stroma (tunica albuginea).

**Figure 3:**
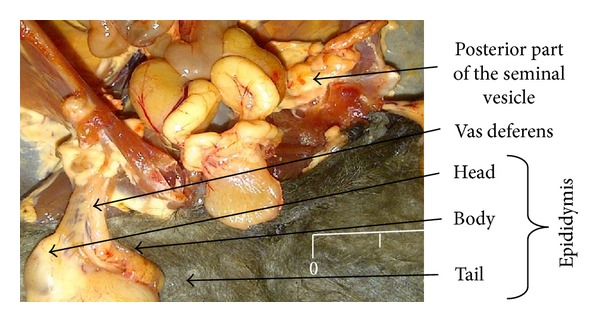
Epididymis of Bat showing the 3 main segments (head, body, and tail) and vas deferens.

The epididymis was observed to be an elongated and convoluted tube that lies firmly attached laterally to the testis. It covers the superior pole of the testis and connects by a narrow corpus epididymis to a prominent cauda epididymis projecting from the distal border of the testis. Macroscopically, the three main segments, caput (head), corpus (body), and cauda (tail), were observed (Figure 3).

Qualitatively, the cauda epididymis was found more enlarged and highly coiled in the adult and was found to be in a straightened tubular-like form in the juvenile.

**Figure 4:**
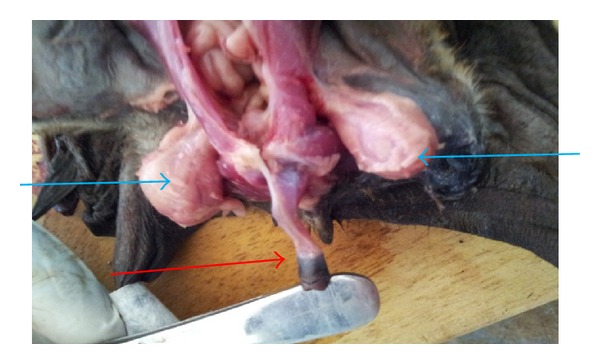
Penis of bat showing its cylindrical nature (red arrow) and testis (blue arrow).

The vas deferens which is a continuation of the cauda epididymis is more straightened and tubular and was observed to be more muscular than the epididymis (Figure 4).

The penis was observed as a cylindrical organ suspended from the pubic arch. It is broader proximally but tapers distally and projected cranially. It takes origin as two crurawhich unite beneath the pubic symphysis near its cranial margin (Figure 4).

Table 2 shows the mean testicular weight and length of different parts of reproductive tract in both adult and juvenile bats with no significant difference (*P* > 0.05) between the right and left testicular weights and between their sizes (length, breath, and circumference) and also between right and left epididymes and vas deferens.

No significant difference (*P* > 0.05) was observed between the penile length of the adult and juvenile (Table 2).

## 4. Discussion

The body mass obtained was in line with the findings of [20], who reported a range of mean body mass of 230–330 g in adult male. The length of the forearm as observed in this study does not differ in any way from the findings of [20] who obtained a mean length of forearm of 117 to 132.0 mm in adult male. The work in [21] reported that there is strong direct relationship between the reproductive status and body condition index and that the body condition index is very important for gonadal development.

As observed from these studies, the bat reproductive tract has great similarity to that of other mammals. This finding is in agreement with report of [22], that gross structures of the reproductive organs in bat follows the normal mammalian pattern. Morphologically, the male reproductive tract is constituted by the testis, epididymis, vas deferens, and penis and this was also reported by [22].

In this study the shape and colouration of the testes were in agreement with the report of [23], in* Cynopterus sphinx gangeticus* bat, that the testes were ovoid in shape with creamy to milky-whitish colouration, whereas the location of the testis which is in the scrotum was contrary to that of [23], who reported that the testes of* Cynopterus sphinx gangeticus* were found in the abdominal region at the base of the urinary bladder while the scrotal sac or pouch is absent. This difference could result from genetic variation between species.

Our findings in this research are similar to the report of [24] in* Taphozous hilli* bat in which the testes were found in the scrotum connected by spermatic cord via the inguinal canal where they appeared to lie within nonpigmented fascial sac which extends from external inguinal ring. This finding is also contrary to the findings of [25] in* Rhinolophus megaphyllus* bat in which the testeslies are just external to the external inguinal ring. The testicular location or position has a significant effect on spermatogenesis in bats [22]. In cases where the testes are intra-abdominal, spermatogenesis ceases entirely [22]. All the variation in terms of the position and location of the testes seen could be attributed to the reproductive activity in adults. The work in [22] also suggested that the present scrotal sac serves to reduce testicular temperature to the level below that of the body temperature.

The division of epididymis into three regions, caput, corpus, and cauda regions, was similar to studies of [22] in male reproductive system of the bat and also in line with the findings of [26], that 3 and 4 segments were found in* Eumops glaucinus *and* Molossus molossus* bats respectively, namely, initial, caput, corpus, and cauda segments. But this division into segments could be attributed to species specificity. The shape of the cauda epididymis in the adult is more conspicuous than in juvenile and this is in line with the report of [22] who reported that the conspicuous nature of the cauda epididymis is a more appropriate indicator of sexual maturity than colour as reported by [27]. These findings were similar to that of [28], who reported that cauda epididymis in young* Plecotus auritus *is small and inconspicuous compared to those in the adult male group.

The morphological presentation of the penis, which was observed to be cylindrical and directed cranially, agrees with [22], who reported that taxonomic variation exists in the posture of the penis which may be directed either cranially or caudally. It was, however, further stated that the morphological architecture is essentially the same in all species of bats.

*Morphometry.* The mean dimension of testes observed in this study was contrary to the findings of [29], who reported 20 by 18 mm in adult male bat. The change observed in testicular size agrees with the findings of [19, 30], who reported that maximum testicular size is associated with maximal spermatic activity in the adult bats. The increase in testicular mass and dimension as seen in these studies has a great influence and role to play in male spermatogenesis as reported by [31, 32]. The epididymal length in adult of this study was similar to the report of [33], who reported 2.5 cm as the epididymal length in adult* Myotis capaccinii* bat in Iran. The epididymal length plays a role in sperm cell storage and reducing sperm cell motility. Whereas the length of the vas deferens observed was contrary to the findings of [34] that recorded 20 cm in humans. This finding could be linked to the distance and location of the testis to the ampullary gland in humans.

The length (3.38 ± 0.18 cm) of the penis in this study was higher than the value obtained by [35] who recorded 1.2 cm in captive* Desmodus rotundus* bat.

## 5. Conclusion

In conclusion, this work is probably the first gross and morphometric studies on the male reproductive system of the bat (*Eidolon helvum*) in Nigeria and has established a baseline data for the different segments of the male reproductive tract of the bat. It is expected that the results will guide further researches on the reproductive systems of the male bats.

## Recommendation

Seasonal variation could be looked at in respect to the environment of the location. It is recommended that the histochemical and ultrastructural studies of the male reproductive tract are recommended for further insight into the reproductive biology of the species.

## Figures and Tables

**Table 1:** Mean body weight (g) and forearm length (cm) of adult and juvenile bats.

	Adult (*n* = 17)	Juvenile (*n* = 13)
Body weight (g)	235.31 ± 6.30	69.54 ± 7.68
Forearm length (cm)	12.14 ± 0.19	7.77 ± 0.29

The values are expressed as mean ± SEM.

*n*: number of bats used.

**Table 2:** Mean testicular weight and length of various parts of reproductive tract in both adult and juvenile bats.

	Adult (*n* = 17)		Juvenile (*n* = 13)	
	Right	Left	Right	Left
TW (g)	1.93 ± 0.1	1.90 ± 0.10	1.42 ± 0.10	1.41 ± 0.10
TL (cm)	1.39 ± 0.09	1.39 ± 0.08	1.10 ± 0.07	1.13 ± 0.07
TB (cm)	1.02 ± 0.05	1.06 ± 0.07	0.89 ± 0.07	0.96 ± 0.07
TC (cm)	2.68 ± 0.20	2.67 ± 0.17	2.45 ± 0.12	2.49 ± 0.11
EL (cm)	2.78 ± 0.17	2.80 ± 0.14	2.43 ± 0.25	2.40 ± 0.22
VDL (cm)	3.69 ± 0.18	3.65 ± 0.14	2.98 ± 0.24	2.92 ± 0.23
PL (cm)	3.38 ± 0.18		3.26 ± 0.13	

The values are expressed as mean ± SEM. No significant difference, *P* > 0.05. TW: testicular weight, TL: testicular length, TB: testicular breadth, TC: testicular circumference, EL: epididymal length, VDL: vas deferens length, PL: penile length.
